# Noise-enabled optical ratchets

**DOI:** 10.1038/srep44287

**Published:** 2017-03-13

**Authors:** Roberto de J. León-Montiel, Pedro A. Quinto-Su

**Affiliations:** 1 Instituto de Ciencias Nucleares, Universidad Nacional Autónoma de México, Apartado Postal 70-543, 04510 Cd. Mx., México

## Abstract

In this contribution, we report on the implementation of a novel noise-enabled optical ratchet system. We demonstrate that, unlike commonly-used ratchet schemes—where complex asymmetric optical potentials are needed—efficient transport of microparticles across a one-dimensional optical lattice can be produced by introducing controllable noise in the system. This work might open interesting routes towards the development of new technologies aimed at enhancing the efficiency of transport occurring at the micro- and nanoscale, from novel particle-sorting tools to efficient molecular motors.

Noise has long been considered as detrimental for energy transport in complex systems. However, recently it has been shown that for certain coherently evolving systems, noise can indeed enhance their transport efficiency^1^,^2^,^3^,^4^,^5^,^6^. This fascinating phenomenon, coined environment-assisted quantum transport, has been experimentally observed in systems where controllable noise has been introduced in order to enhance the transfer efficiency of electronic^7^ and optical^8^,^9^,^10^ signals. Interestingly, recent theoretical studies have suggested that this effect may be observed in purely classical systems^11^ and that it might be exploited to enhance the transport of microscopic objects across potential energy landscapes^12^,^13^,^14^.

Transport across an array of potentials has been achieved in the micro- and nanoscale domain by means of ratchet systems, where movement of a particle is mediated by a combination of a periodic external force and asymmetric potentials which privilege motion in one direction while hindering it in the opposite^15^,^16^,^17^,^18^,^19^,^20^,^21^,^22^. These asymmetric potentials represent the ratchet and the pawl in the classical Smoluchowski-Feynman ratchet^23^,^24^,^25^, while the periodic force represents the Brownian perturbations. There is also the possibility of symmetric potentials synchronized with an external force, which breaks the temporal (rather than spatial) symmetry of the system producing directed motion^26^,^27^,^28^.

In this paper, we report on the transport of a single microparticle across a one-dimensional symmetric optical lattice, induced by dynamical disorder or noise. The optical potentials are created by focused beams (optical tweezers) that trap the microparticle in three dimensions. Movement of the particle is then enabled by introducing random fluctuations in the power of each individual trap, changing the depth of the potentials at a fixed frequency, *f*, which can take different values between 0 and 35 Hz. In our setup, directed motion of the particle is guaranteed by introducing a weak external force in the system. This force is smaller than the one necessary to make the particle escape the potentials and hence it is not sufficient to create transport by itself. This system resembles a tilted Smoluchowski-Feynman ratchet^29^, where a constant external force is added to the potentials, slightly tilting them in the direction of the force.

## Results

### Model

**Figure 1:**
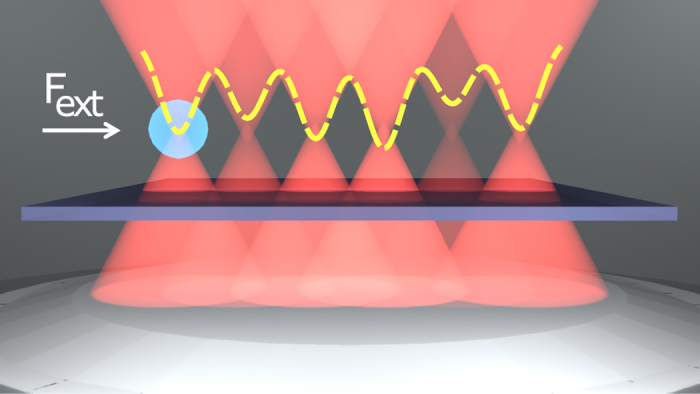
Schematic representation of the optical tweezer array comprising six equally-spaced focused beams affected by noise. The dashed line represents the Gaussian approximation of the potentials, as described in Eq. (1). Notice that the effect of introducing dynamical disorder (noise) in the power of each trap is to effectively change the depth of individual potentials, which ultimately leads to the directed motion of the particle.

We model the ratchet system as a Brownian particle moving in a dynamically-disordered one-dimensional potential landscape (Fig. 1) comprising *N* closely-spaced Gaussian potential wells of the form^30^,^31^


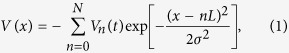


where *V*_*n*_(*t*) and *σ* stand for the depth and width of the wells, and *L* is the separation between them. Because in the experiment noise is introduced as random changes in the power at each trap (trap depth), fluctuations in the depth of the optical wells may be described as


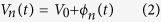


with *V*_0_ being the mean depth of the wells and *ϕ*_*n*_(*t*) a Gaussian Markov process with zero average, i.e. 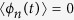 and 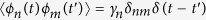, where 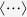 denotes stochastic averaging.

The motion of the Brownian particle in a potential such as the one in Eq. (1) can be well described by the Langevin equation, in the overdamped limit^32^, as


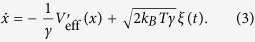


Here, *γ* characterizes the friction of the particle immersed in liquid, and 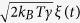 the thermal noise due to random collisions with the surrounding fluid molecules. *ξ*(*t*) describes a Gaussian stochastic process with zero average and a white-noise autocorrelation function^32^, *k*_*B*_ is the Boltzmann constant, and *T* the temperature of the system. Notice that, in Eq. (3), we have defined an effective potential 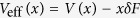, where *δF* is a weak, constant external force. Because of its construction, one may identify Eq. (3) as a tilted Smoluchowsky-Feynman ratchet^29^, with the important difference that, in our system, the perturbation *δF* is used only to guarantee a directed motion of the particle and not to create transport by itself.

### Experiment

The ratchet is experimentally implemented in a standard holographic optical tweezer setup, with a trapping laser of wavelength *λ* = 1064 nm. In our experiments, we make use of a single trapped microparticle of mean diameter 2*R* = 2.50 ± 0.1 *μ*m. The particle is taken from a colloidal sample consisting of water with silica microbeads (Bangs Laboratories), which is contained within two microscope coverslips (No. 1, 0.13–0.16 mm thick) separated by ~100 *μ*m. The optical potentials are created by a two-dimensional phase hologram introduced in the path of the trapping beam by means of a spatial light modulator (SLM, Holoeye Pluto NIR-2). The structured laser beam is then simultaneously focused at six spots with a spatial separation of 1.88 ± 0.05 *μ*m by a 100x microscope objective (NA 1.25) in a custom inverted microscope with a piezo-electric stage. The total diffracted power, transmitted by the microscope objective, into all of the potentials is measured to be *P* = 26 mW.

**Figure 2:**
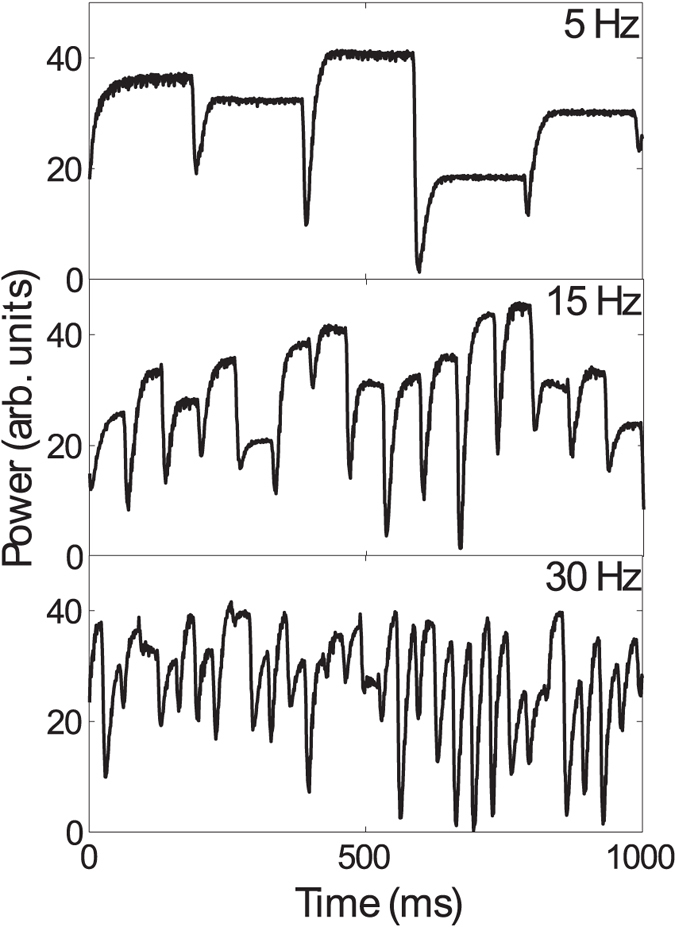
Measured power at one trap at 1000 fps for noise at a frequency *f* = 5, 20 and 30 Hz.

In our scheme, noise is introduced in the system as random fluctuations in the optical power of individual traps, at a frequency between 0 and 35 Hz with a standard deviation of 30%. This is implemented by projecting different phase holograms into the SLM, at the corresponding frequency, where the amplitudes of the optical potentials are randomly changed while keeping the mean diffracted power constant (see Methods for details). Figure 2 shows the integrated power at one trap driven at different frequencies. Notice that each time the frame changes there is flickering, which results in a lowering of diffracted power to the trap. We have addressed this issue by adjusting the laser power, while choosing a proper hologram set that yields the correct standard deviation (see Methods for details).

Finally, the constant external force is introduced by dragging the piezo-electric stage at a constant rate of 10 *μ*m/s. Notice that the force experienced by the spherical particle can be obtained by means of the Stokes expression *F*_*d*_ = 6*πηRv*, where *η* is the liquid viscosity and *v* is the drag speed. In our experiment, the drag has to be corrected, as the particle is near the bottom boundary at a height *h* = 16 *μ*m, with the Faxen correction, which to the third order of *r* = *R*/*h* yields 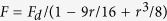
33, and has a contribution of +4% compared to the Stokes expression. Here, it is important to remark that the force necessary to leave the potentials is given by the escape velocity. The mean escape velocity for different patterns with the highest uniformity was measured to be 52 ± 12 *μ*m/s. Therefore the external force of 10 *μ*m/s is not enough to induce transport, even for the potential wells that have the lowest power when noise is introduced in the system. Also, notice that the flickering effect, where for some events the intensity drops to very low values, allows the particle to be essentially free for about 5 ms. This may result in a displacement of 50 nm (for a drag speed of 10 *μ*m/s) which is again not enough to reach a neighboring potential well.

**Figure 3:**
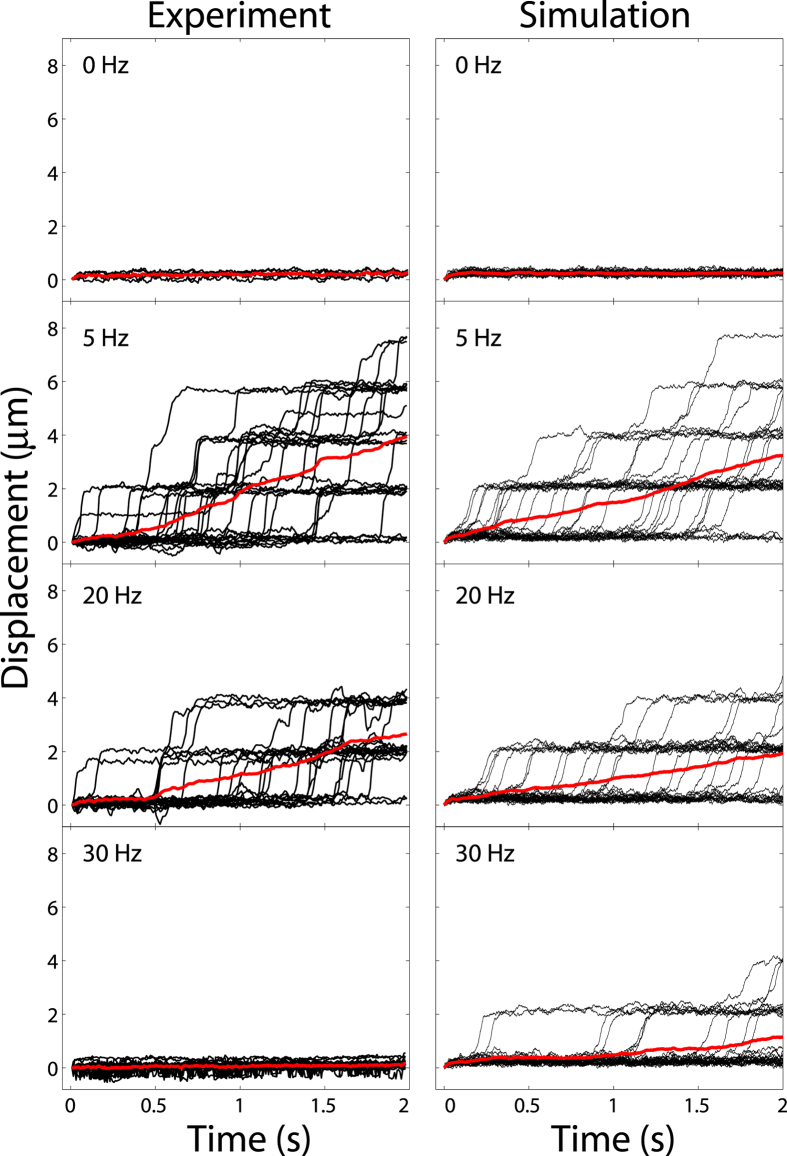
Microparticle trajectories for noise at *f* = 0, 5, 20 and 30 Hz. Left column: Experiment. Right column: Simulation. The red line represents the average trajectory over 30 different realizations.

The experiments were performed using noise frequencies *f* between 0 and 35 Hz, and the dynamics of the microparticle were recorded at 125 fps during 60 seconds for each *f* value. A sample of the measured trajectories for selected frequencies are shown in the left column of Fig. 3. The right column shows the simulated trajectories for the same noise frequencies using the model described by Eq. (3). In both columns, the thick red line represents the average over all trajectories. We observe that, as expected, there is no transport for a static pattern since the drag speed is much smaller than the escape velocity. However, as the noise frequency increases, the particle starts to diffuse across the potentials following an average path characterized by a constant speed (ballistic transport) that reaches its optimum value (~2.3 *μ*m/s) at 5 Hz for the experiment and simulations. Under the conditions of this study it is important to highlight that the optimum transport frequency does not depend on the magnitude of the external force as long as it is much smaller than the escape velocity. Finally, as noise becomes larger, transport of the particle decreases and stops at 30 Hz in the experiment, whereas the simulation still shows a very low transport speed.

## Discussion

**Figure 4:**
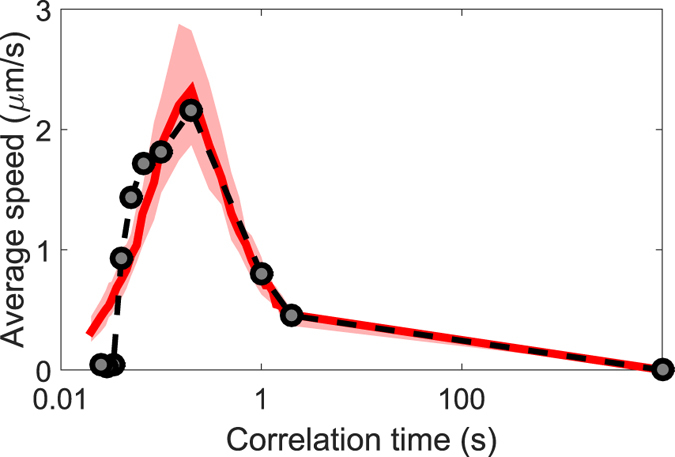
Average speed of the particle as a function of the noise correlation time *τ* = 1/*f*. Experimental results: round symbols (dotted line to guide the eye), were obtained by averaging the speed of the particle over 30 realizations. The red solid line represents the simulated average speed of the particle numerically obtained for the same number of realizations. The shaded region represents the average speed of the particle considering the ±0.1 *μ*m uncertainty in the particle’s mean diameter.

Figure 4 shows the measured average transport speeds (round symbols) as a function of the noise correlation time, which we define as the inverse of the noise frequency *τ* = 1/*f*. The continuous red line represents the results for the simulations which agree reasonably well in the whole range with a small discrepancy at the highest frequencies. As pointed out by previous authors, whether noise-assisted transport occurs depends on a competition of time scales^34^,^35^. In particular, we can compare the configurational relaxation time of the particle—the time 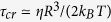 needed for the particle to diffuse across its own radius^36^,^37^—and the noise correlation time *τ*. At short correlation times (high frequencies), the optical potentials change so fast, compared to the dynamics of the system, that the particle does not “feel” any change in the energy landscape. In this case, the particle is likely to remain trapped in each realization, thus leading to a small average speed of about 0.4 *μ*m/s. When increasing the correlation time *τ*, the average speed of the particle grows up to 2.3 *μ*m/s. Interestingly, the maximum value of the particle’s average speed is reached when the noise correlation time is approximately the same as the configurational relaxation time of the particle, 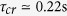. This is due to the fact that, in our experiment, the separation between optical traps is ~1.5 *R*, which implies that when the particle travels a distance *R* (direction set by external force) it has traveled more than half the distance to the next potential, so that if at time *τ*_*cr*_ the potentials change it is possible that the depth of the potential that is now closer to the particle is larger, thus effectively pulling the particle in the direction of the external force. Finally, for larger correlation times, the average speed of the particle drops rapidly to zero. This is because the noise correlation time becomes so large that the optical potentials do not change during each measurement, resulting in a system that is no longer affected by noise.

To conclude, noise-assisted transport has been previously explained as the suppression of coherent quantum localization through noise, bringing the detuned quantum levels into resonances and thus facilitating energy transfer^3^,^5^. The results presented here show that this phenomenon can be observed in a broader class of systems, even the macroscopic ones. This opens interesting routes towards new methods for enhancing the efficiency of the ratchet mechanism, from particle sorting systems to efficient molecular motors. In this way, a phenomenon initially conceived in a quantum scenario has shown to apply as well in classical macroscopic systems, widening the scope of possible quantum-inspired technological applications.

## Methods

The digital holograms that generate the six periodic optical potentials are calculated with the Gerchberg-Saxton algorithm (GS)^38^, and the weighted GS algorithm^39^. Noise is introduced by randomly changing holograms with different standard deviations between 5 and 40%, while keeping the mean power constant. The intensity for each resulting trap array is measured by means of a high speed camera (Photron SA 1.1), which records the dynamics of the back reflected beam at 1000 fps. The holograms are then classified by the measured uniformity in steps of 5%. In this way, we create videos by arranging the calculated holograms randomly at each uniformity at frame rates between 0.5 to 35 Hz.

As we discussed above, when introducing noise in the system, the power of individual traps exhibits flickering, which results in a lowering of the diffracted power. We have addressed this issue by characterizing the refreshing process of the SLM at the frequencies of interest. The measured characteristic rise/fall times (defined at 10–90% of the transition) of the SLM are: 23.9 ± 5.3 ms and 4.9 ± 1.2 ms respectively. In addition, we found that the time where the optical power of the traps is below 10% is 4.6 ± 2.2 ms, and that the power dropped to less than 1/4 of the mean for 36% of the transitions. From these numbers, one can find that the time to reach a stationary value of the intensity, after switching a hologram, is about 30 ms. This corresponds to a frequency of 33 Hz, which is close to an upper bound for the noise frequency. Notice that as the frequency of noise increases, the flickering time becomes comparable to the period of the cycle, so that while the intensity is rising the pattern switches before it can reach the stationary value, effectively lowering the mean power delivered to the trap. This has also the effect of rising the standard deviation of the time series for the optical power at each trap. Therefore, videos at the highest frame rates of 35 Hz with the highest uniformity in the holograms yield the lower boundary for the standard deviation which is about ±30%. In this way, at every video frame rate (or noise frequency) we choose the hologram set that yields a variation of ±30% while adjusting the diffracted power to keep the mean power in the time series constant at all frequencies.

## Additional Information

**How to cite this article:** León-Montiel, R. de. J. and Quinto-Su, P. A. Noise-enabled optical ratchets. *Sci. Rep.* **7**, 44287; doi: 10.1038/srep44287 (2017).

**Publisher's note:** Springer Nature remains neutral with regard to jurisdictional claims in published maps and institutional affiliations.
